# The genome sequence of the amphioxus, *Branchiostoma lanceolatum *(Pallas, 1774)

**DOI:** 10.12688/wellcomeopenres.23671.1

**Published:** 2025-02-24

**Authors:** Patrick Adkins, John Bishop, Joanna Harley, Peter W. H. Holland, Marine Biological Association Genome Acquisition Lab, Darwin Tree of Life Barcoding collective, Wellcome Sanger Institute Tree of Life Management, Samples and Laboratory team, Wellcome Sanger Institute Scientific Operations: Sequencing Operations, Wellcome Sanger Institute Tree of Life Core Informatics team, Tree of Life Core Informatics collective, Darwin Tree of Life Consortium

**Affiliations:** 1 The Marine Biological Association, Plymouth, England, UK; 2 University of Oxford, Oxford, England, UK

**Keywords:** Branchiostoma lanceolatum, amphioxus, genome sequence, chromosomal, Amphioxiformes

## Abstract

We present a genome assembly from a specimen of *Branchiostoma lanceolatum* (Amphioxus; Chordata; Leptocardii; Amphioxiformes; Branchiostomatidae). The assembly contains two haplotypes with total lengths of 468.40 megabases and 465.81 megabases, respectively. Most of haplotype 1 (99.34%) is scaffolded into 19 chromosomal pseudomolecules. Haplotype 2 is a scaffold level assembly. The mitochondrial genome has also been assembled and is 15.14 kilobases in length.

## Species taxonomy

Eukaryota; Opisthokonta; Metazoa; Eumetazoa; Bilateria; Deuterostomia; Chordata; Cephalochordata; Leptocardii; Amphioxiformes; Branchiostomatidae; *Branchiostoma*; *Branchiostoma lanceolatum* (Pallas, 1774) (NCBI:txid7740)

## Background

Lancelets or amphioxus are benthic filter-feeding marine invertebrates belonging to the subphylum Cephalochordata in the Phylum Chordata. Active animals capable of rapid movement, amphioxus have a stiff notochord running the length of the body, a dorsal nerve cord, pharyngeal slits and lateral segmented muscle blocks. These characters probably date back to the earliest chordates leading E.S. Goodrich to note “ *Amphioxus is doubtless in some respects a very specialized animal . . . yet it preserves many primitive characters*” ( Gaskell *et al.*, 1910). Several amphioxus species have been studied intensively as morphologically simple chordates and as outgroups for understanding the evolutionary origins of the vertebrates ( D’Aniello *et al.*, 2023; Holland & Holland, 2022). These comparisons extend to the molecular level, with cephalochordates often retaining single-copy genes homologous to the multigene families of vertebrates generated by genome duplications ( Furlong & Holland, 2002; Holland *et al.*, 2008).

There are over 20 species of cephalochordate worldwide but only one species, *Branchiostoma lanceolatum*, is recorded from Britain and Ireland. *B. lanceolatum* can be locally abundant in sand and gravel sediments off the coast of Devon and Cornwall, with scattered records from shallow seabed habitats around England, Wales and Scotland as far north as Shetland ( NBN Atlas Partnership, 2024). A large population living in coarse shell gravel at 20 to 60 metres depth off the Eddystone reef south of Plymouth has been studied for over a century ( Allen, 1899; Smith, 1932). There are also numerous records of planktonic amphioxus larvae sampled in Continuous Plankton Recorder surveys in the North Sea and Irish Sea ( NBN Atlas Partnership, 2024), but these are difficult to identify to species level and the source populations not known. *B. lanceolatum* has a wide distribution in Europe, from the North Sea to the Atlantic coast of France and Portugal, and along the Mediterranean coastline of Spain, France and Italy ( GBIF Secretariat, 2023). There are also records of *B. lanceolatum* from the Red Sea and Arabian Sea, possibly spread through the Suez Canal, but species identification has not been verified genetically ( Maghsoudlou, 2018). Differences in spawning period, developmental rate and adult body size between Atlantic and Mediterranean populations raised questions over whether these are the same species; analyses of mitochondrial DNA sequences strongly support conspecificity ( Caccavale *et al.*, 2021). The genetic or environmental basis for these morphological and life-history differences are not known, and most genetic work has been conducted on Mediterranean *B. lanceolatum* rather than on Atlantic populations.

Two high-quality genome sequences have been published from individual *B. lanceolatum* specimens collected in the Mediterranean Sea close to Argeles-sur-Mer, France ( Brasó-Vives *et al.*, 2022; Marlétaz *et al.*, 2018). Here we report the complete genome sequence of an individual female *B. lanceolatum* collected from coastal sediments off Devon, UK. The genome sequence reported here will facilitate research into population genetics and local adaptations of *B. lanceolatum* in northern European waters and enable comparative analyses between Atlantic and Mediterranean *B. lanceolatum*. The genome sequence adds to a growing genomic data set for cephalochordates, a group of animals key for understanding the origins of vertebrates.

## Genome sequence report

**Figure 1.**
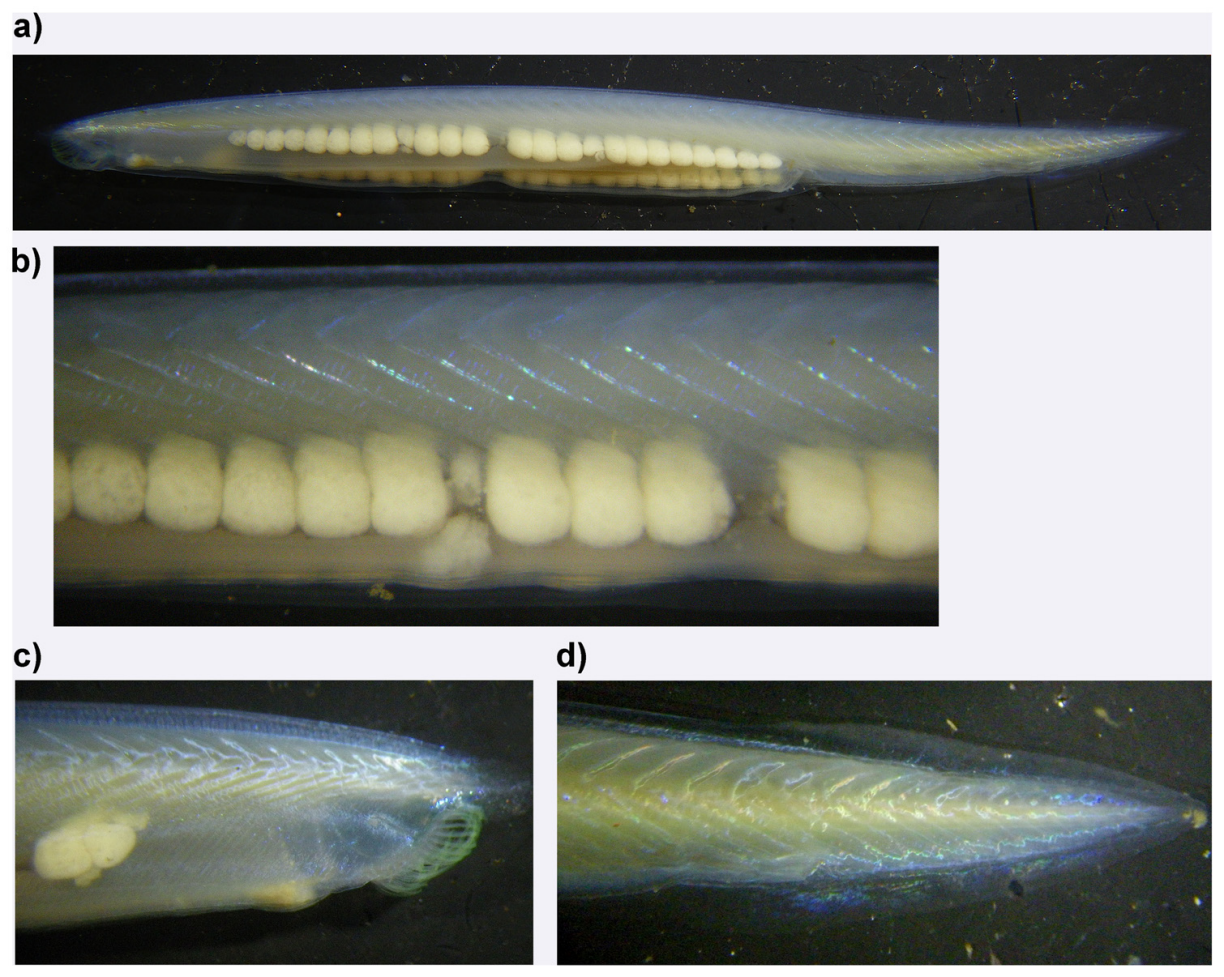
Photographs of the *Branchiostoma lanceolatum* (klBraLanc2) specimen used for genome sequencing. (a) Lateral view, anterior to the left. (b) Close-up of ripe gonads. (c) Close up of anterior showing oral cirri and pharyngeal slits. (d) Close up of tail showing chevron shaped lateral muscle blocks.

The genome of an adult *Branchiostoma lanceolatum* ( Figure 1) was sequenced using Pacific Biosciences single-molecule HiFi long reads, generating a total of 35.02 Gb (gigabases) from 2.97 million reads, providing approximately 75-fold coverage. Primary assembly contigs were scaffolded with chromosome conformation Hi-C data, which produced 103.82 Gb from 687.58 million reads. Specimen and sequencing details are provided in Table 1.

**Table 1.** Specimen and sequencing data for *Branchiostoma lanceolatum*.

Project information			
**Study title**	Branchiostoma lanceolatum (amphioxus)		
**Umbrella BioProject**	PRJEB74948		
**Species**	*Branchiostoma lanceolatum*		
**BioSample**	SAMEA111562176		
**NCBI taxonomy ID**	7740		
Specimen information			
**Technology**	**ToLID**	**BioSample accession**	**Organism part**
**PacBio long read sequencing**	klBraLanc2	SAMEA111562385	muscle
**Hi-C sequencing**	klBraLanc2	SAMEA111562383	muscle
**RNA sequencing**	klBraLanc2	SAMEA111562386	muscle
Sequencing information			
**Platform**	**Run accession**	**Read count**	**Base count (Gb)**
**Illumina NovaSeq 6000 (Hi-C)**	ERR12945456	6.88e+08	103.82
**Revio (PacBio)**	ERR12921304	2.97e+06	35.02
**Illumina NovaSeq X (RNA)**	ERR13493941	8.88e+07	13.41

The two haplotypes were combined for curation. Manual assembly curation corrected 51 missing joins or mis-joins and 22 haplotypic duplications. This reduced the scaffold number by 21.79%.

**Figure 2.**
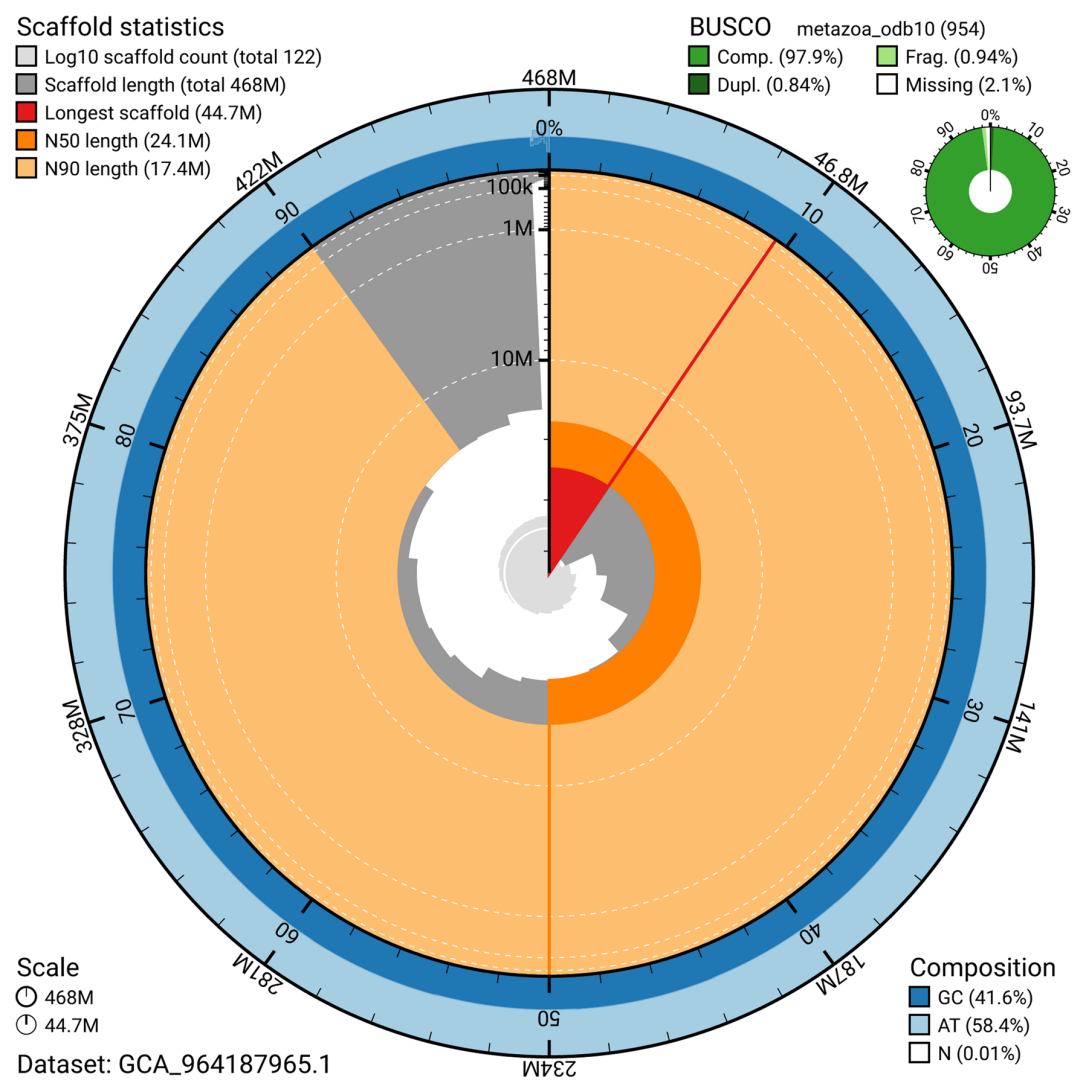
Genome assembly of *Branchiostoma lanceolatum*, klBraLanc2.hap1.1: metrics. The BlobToolKit snail plot provides an overview of assembly metrics and BUSCO gene completeness. The circumference represents the length of the whole genome sequence, and the main plot is divided into 1,000 bins around the circumference. The outermost blue tracks display the distribution of GC, AT, and N percentages across the bins. Scaffolds are arranged clockwise from longest to shortest and are depicted in dark grey. The longest scaffold is indicated by the red arc, and the deeper orange and pale orange arcs represent the N50 and N90 lengths. A light grey spiral at the centre shows the cumulative scaffold count on a logarithmic scale. A summary of complete, fragmented, duplicated, and missing BUSCO genes in the metazoa_odb10 set is presented at the top right. An interactive version of this figure is available at https://blobtoolkit.genomehubs.org/view/Branchiostoma_lanceolatum/dataset/GCA_964187965.1/snail.

**Figure 3.**
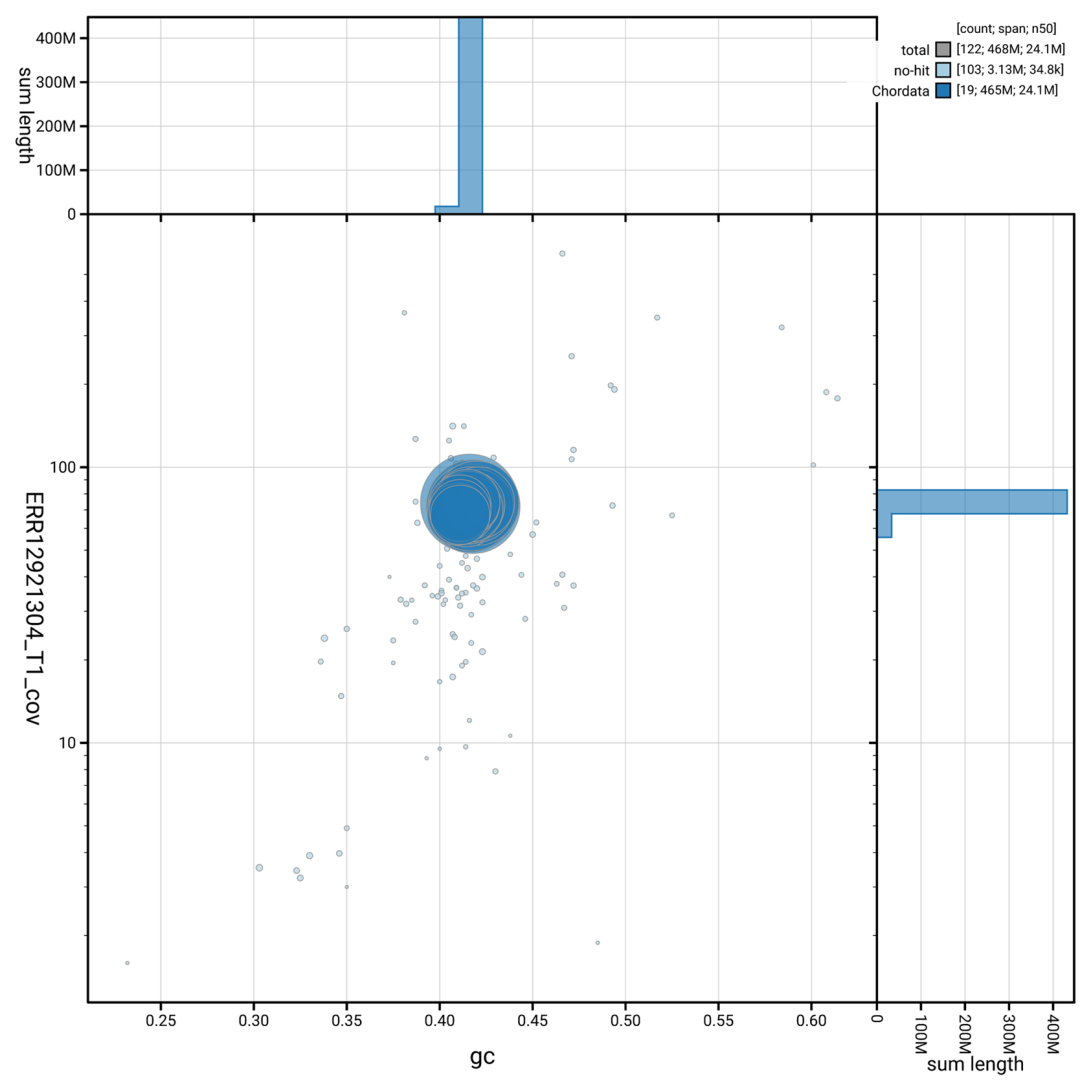
Genome assembly of *Branchiostoma lanceolatum*, klBraLanc2.hap1.1: BlobToolKit GC-coverage plot. Blob plot showing sequence coverage (vertical axis) and GC content (horizontal axis). The circles represent scaffolds, with the size proportional to scaffold length and the colour representing phylum membership. The histograms along the axes display the total length of sequences distributed across different levels of coverage and GC content. An interactive version of this figure is available at https://blobtoolkit.genomehubs.org/view/Branchiostoma_lanceolatum/dataset/GCA_964187965.1/blob.

**Figure 4.**
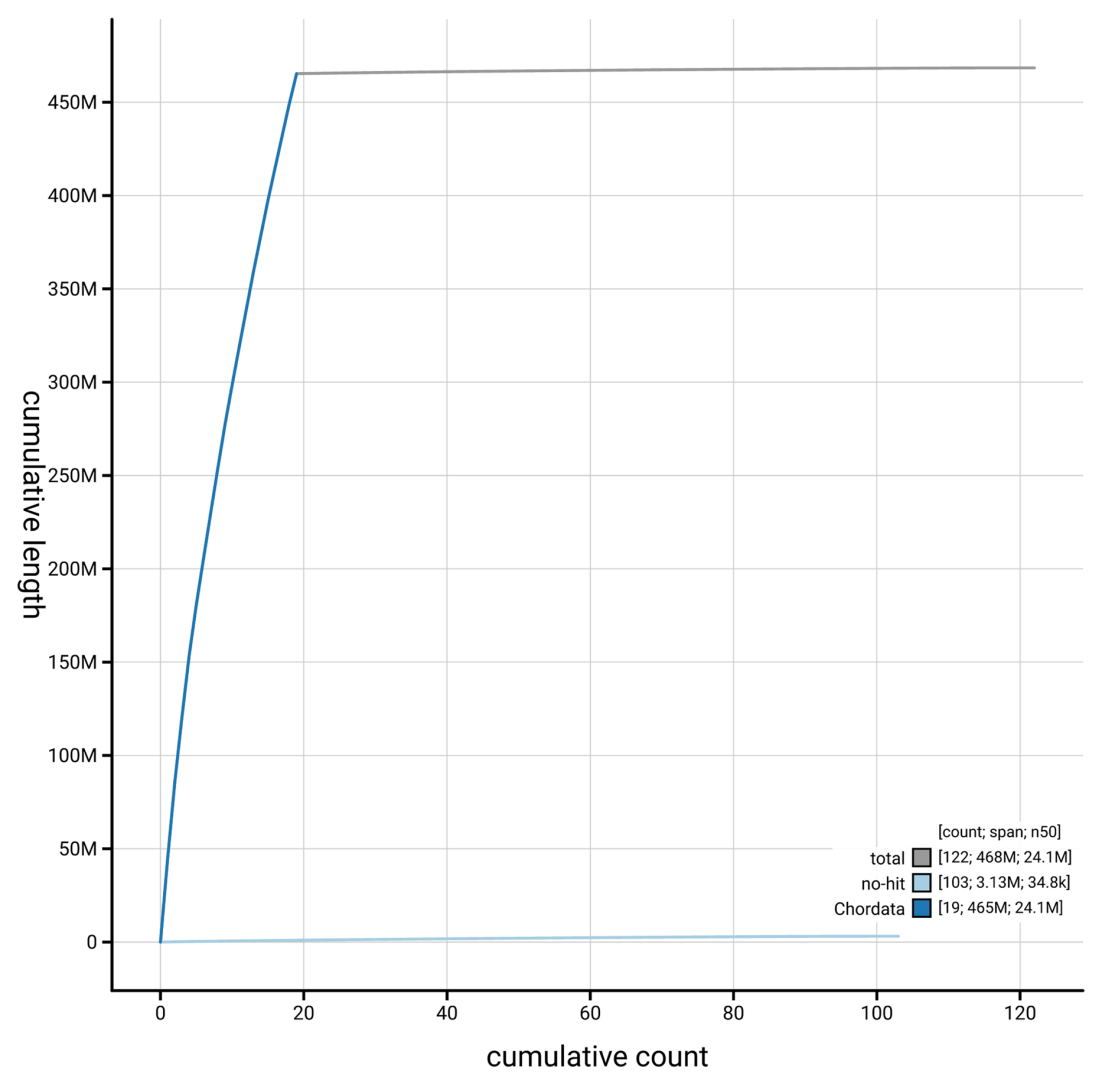
Genome assembly of *Branchiostoma lanceolatum* klBraLanc2.hap1.1: BlobToolKit cumulative sequence plot. The grey line shows cumulative length for all scaffolds. Coloured lines show cumulative lengths of scaffolds assigned to each phylum using the buscogenes taxrule. An interactive version of this figure is available at https://blobtoolkit.genomehubs.org/view/Branchiostoma_lanceolatum/dataset/GCA_964187965.1/cumulative.

**Figure 5.**
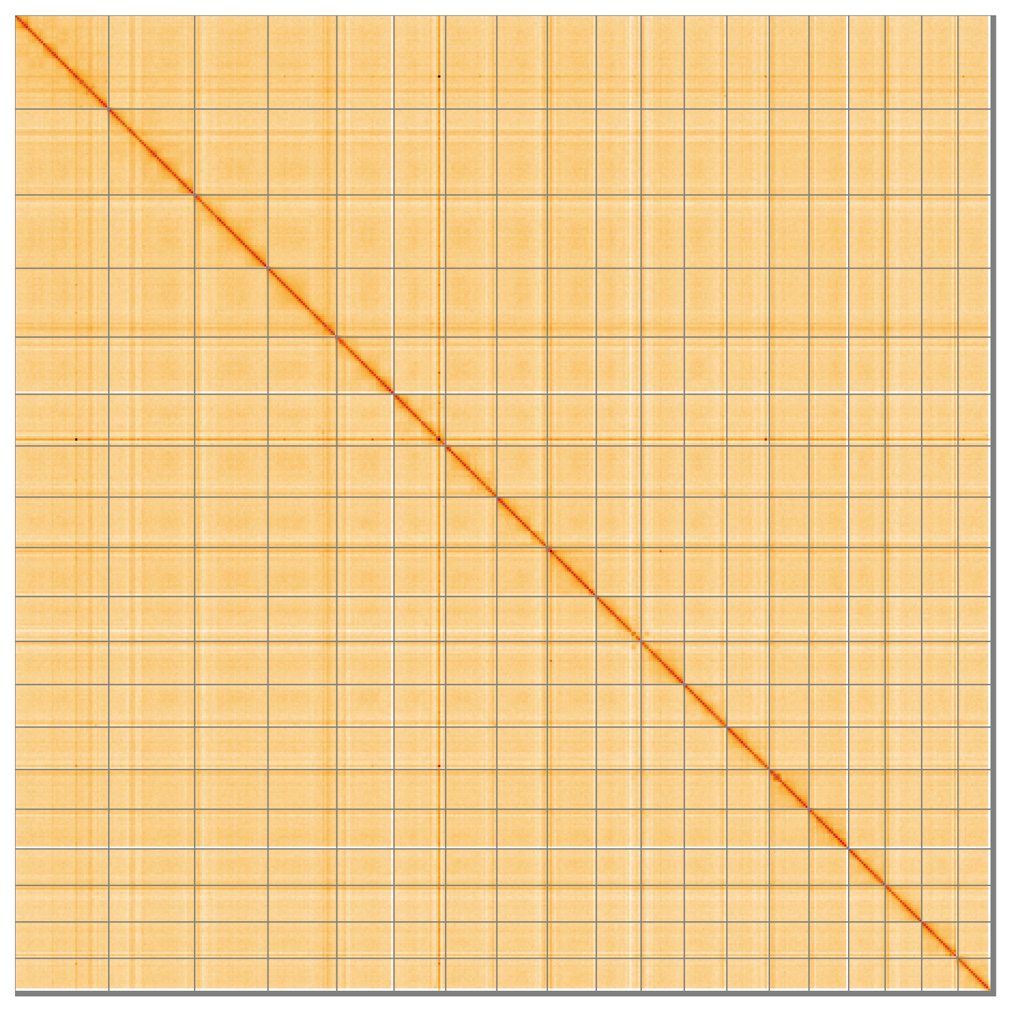
Genome assembly of *Branchiostoma lanceolatum* klBraLanc2.hap1.1: Hi-C contact map of the klBraLanc2.hap1.1 assembly, visualised using HiGlass. Chromosomes are shown in order of size from left to right and top to bottom. An interactive version of this figure may be viewed at https://genome-note-higlass.tol.sanger.ac.uk/l/?d=TDyQj19wQX28exeWeDlF_Q.

The final haplotype 1 assembly has a total length of 468.40 Mb in 121 sequence scaffolds, with 211 gaps, and a scaffold N50 of 24.11 Mb ( Table 2). The snail plot in Figure 2 provides a summary of the assembly statistics, while the distribution of assembly scaffolds on GC proportion and coverage is shown in Figure 3. The cumulative assembly plot in Figure 4 shows curves for subsets of scaffolds assigned to different phyla. Most (99.34%) of the assembly sequence was assigned to 19 chromosomal-level scaffolds. Chromosome-scale scaffolds confirmed by the Hi-C data are named in order of size ( Figure 5; Table 3). 

**Table 2.** Genome assembly data for the *Branchiostoma lanceolatum* assembly.

Genome assembly	Haplotype 1	Haplotype 2
Assembly name	klBraLanc2.hap1.1	klBraLanc2.hap2.1
Assembly accession	GCA_964187965.1	GCA_964188055.1
Assembly level	chromosome	scaffold
Span (Mb)	468.4	465.8
Number of contigs	332	288
Number of scaffolds	121	100
Longest scaffold (Mb)	44.72	None
Assembly metrics (Benchmark) *	Haplotype 1	Haplotype 2
Contig N50 length (≥ 1 Mb)	3.23 Mb	3.63 Mb
Scaffold N50 length (= chromosome N50)	24.11 Mb	23.57 Mb
Consensus quality (QV) (≥ 40)	66.0	66.4
*k*-mer completeness	61.36%	61.30%
*k*-mer completeness combined (≥ 95%)	99.84%	
BUSCO ** (S > 90%; D < 5%)	C:97.8%[S:97.0%,D:0.8%], F:1.2%,M:1.0%,n:954	C:98.2%[S:97.3%,D:0.9%], F:0.9%,M:0.9%,n:954
Percentage of assembly mapped to chromosomes (≥ 90%)	99.34%	-
Organelles (one complete allele)	Mitochondrial genome: 15.14 kb	*-*

* Assembly metric benchmarks are adapted from Rhie *et al.* (2021) and the Earth BioGenome Project Report on Assembly Standards September 2024.

** BUSCO scores based on the metazoa_odb10 BUSCO set using version 5.4.3. C = complete [S = single copy, D = duplicated], F = fragmented, M = missing, n = number of orthologues in comparison.

**Table 3.** Chromosomal pseudomolecules in the genome assembly of *Branchiostoma lanceolatum*, klBraLanc2, haplotype 1.

INSDC accession	Name	Length (Mb)	GC%
OZ076729.1	1	44.72	41.5
OZ076730.1	2	40.94	42
OZ076731.1	3	34.89	41.5
OZ076732.1	4	32.86	42
OZ076733.1	5	27.24	41.5
OZ076734.1	6	24.62	41.5
OZ076735.1	7	24.36	42
OZ076736.1	8	24.11	41.5
OZ076737.1	9	23.35	42
OZ076738.1	10	21.35	41.5
OZ076739.1	11	20.6	41.5
OZ076740.1	12	20.27	41.5
OZ076741.1	13	20.22	41
OZ076742.1	14	18.91	41.5
OZ076743.1	15	18.91	41.5
OZ076744.1	16	17.47	41
OZ076745.1	17	17.43	41
OZ076746.1	18	17.21	41
OZ076747.1	19	15.82	41
OZ076748.1	MT	0.02	38

The mitochondrial genome was also assembled and is included both as a contig within the multifasta file of the genome submission and as a standalone record in GenBank.

The estimated Quality Value (QV) of the final haplotype 1 assembly is 66.0 and of haplotype 2 it is 66.4. The *k*-mer completeness of haplotype 1 is 61.36%, of haplotype 2 is 61.30%, and of the combined haplotypes is 99.84%. The BUSCO v5.4.3 completeness of haplotype 1 is estimated as 97.8% (single = 97.0%, duplicated = 0.8%), using the metazoa_odb10 reference set ( *n* = 954). For haplotype 2, the BUSCO v5.4.3 completeness is 98.2% (single = 97.3%, duplicated = 0.9%), using the metazoa_odb10 reference set ( *n* = 954).

## Methods

### Sample acquisition and DNA barcoding

An adult specimen of *Branchiostoma lanceolatum* (specimen ID MBA-220429-004A, ToLID klBraLanc2) was collected from H1 (Mv Sepia), Bigbury Bay, Devon, United Kingdom (latitude 50.26, longitude –3.95) on 2022-04-29, using a naturalist dredge deployed from the RV Sepia. The specimen was collected and identified by Patrick Adkins, John Bishop and Joanna Harley (Marine Biological Association) and preserved on dry ice.

The initial identification was verified by an additional DNA barcoding process according to the framework developed by Twyford *et al*. (2024). A small sample was dissected from the specimens and stored in ethanol, while the remaining parts were shipped on dry ice to the Wellcome Sanger Institute (WSI). The tissue was lysed, the COI marker region was amplified by PCR, and amplicons were sequenced and compared to the BOLD database, confirming the species identification ( Crowley *et al.*, 2023). Following whole genome sequence generation, the relevant DNA barcode region was also used alongside the initial barcoding data for sample tracking at the WSI ( Twyford *et al.*, 2024). The standard operating procedures for Darwin Tree of Life barcoding have been deposited on protocols.io ( Beasley *et al.*, 2023).

### Nucleic acid extraction

The workflow for high molecular weight (HMW) DNA extraction at the Wellcome Sanger Institute (WSI) Tree of Life Core Laboratory includes a sequence of core procedures: sample preparation and homogenisation, DNA extraction, fragmentation and purification. Detailed protocols are available on protocols.io ( Denton *et al.*, 2023b). The klBraLanc2 sample was prepared for DNA extraction by weighing and dissecting it on dry ice ( Jay *et al.*, 2023). Tissue from the muscle was homogenised using a PowerMasher II tissue disruptor ( Denton *et al.*, 2023a).

HMW DNA was extracted using the Automated MagAttract v2 protocol ( Oatley *et al.*, 2023a). DNA was sheared into an average fragment size of 12–20 kb in a Megaruptor 3 system ( Bates *et al.*, 2023). Sheared DNA was purified by solid-phase reversible immobilisation, using AMPure PB beads to eliminate shorter fragments and concentrate the DNA ( Oatley *et al.*, 2023b). The concentration of the sheared and purified DNA was assessed using a Nanodrop spectrophotometer and Qubit Fluorometer using the Qubit dsDNA High Sensitivity Assay kit. Fragment size distribution was evaluated by running the sample on the FemtoPulse system.

RNA was extracted from muscle tissue of klBraLanc2 in the Tree of Life Laboratory at the WSI using the RNA Extraction: Automated MagMax™ *mir*Vana protocol ( do Amaral *et al.*, 2023). The RNA concentration was assessed using a Nanodrop spectrophotometer and a Qubit Fluorometer using the Qubit RNA Broad-Range Assay kit. Analysis of the integrity of the RNA was done using the Agilent RNA 6000 Pico Kit and Eukaryotic Total RNA assay.

### Hi-C preparation

Muscle tissue from the klBraLanc2 sample was processed at the WSI Scientific Operations core, using the Arima-HiC v2 kit. Frozen tissue (stored at –80 °C) was fixed, and the DNA crosslinked using a TC buffer with 22% formaldehyde. After crosslinking, the tissue was homogenised using the Diagnocine Power Masher-II and BioMasher-II tubes and pestles. Following the kit manufacturer's instructions, crosslinked DNA was digested using a restriction enzyme master mix. The 5’-overhangs were then filled in and labelled with biotinylated nucleotides and proximally ligated. An overnight incubation was carried out for enzymes to digest remaining proteins and for crosslinks to reverse. A clean up was performed with SPRIselect beads prior to library preparation.

### Library preparation and sequencing

***PacBio HiFi***

At a minimum, samples were required to have an average fragment size exceeding 8 kb and a total mass over 400 ng to proceed to the low input SMRTbell Prep Kit 3.0 protocol (Pacific Biosciences, California, USA), depending on genome size and sequencing depth required. Libraries were prepared using the SMRTbell Prep Kit 3.0 (Pacific Biosciences, California, USA) as per the manufacturer's instructions. The kit includes the reagents required for end repair/A-tailing, adapter ligation, post-ligation SMRTbell bead cleanup, and nuclease treatment. Following the manufacturer’s instructions, size selection and clean up was carried out using diluted AMPure PB beads (Pacific Biosciences, California, USA). DNA concentration was quantified using the Qubit Fluorometer v4.0 (Thermo Fisher Scientific) with Qubit 1X dsDNA HS assay kit and the final library fragment size analysis was carried out using the Agilent Femto Pulse Automated Pulsed Field CE Instrument (Agilent Technologies) and gDNA 55kb BAC analysis kit.

Samples were sequenced on a Revio instrument (Pacific Biosciences, California, USA). Prepared libraries were normalised to 2 nM, and 15 μL was used for making complexes. Primers were annealed and polymerases were hybridised to create circularised complexes according to manufacturer’s instructions. The complexes were purified with the 1.2X clean up with SMRTbell beads. The purified complexes were then diluted to the Revio loading concentration (in the range 200–300 pM), and spiked with a Revio sequencing internal control. Samples were sequenced on Revio 25M SMRT cells (Pacific Biosciences, California, USA). The SMRT link software, a PacBio web-based end-to-end workflow manager, was used to set-up and monitor the run, as well as perform primary and secondary analysis of the data upon completion.

***Hi-C***

For Hi-C library preparation, DNA was fragmented using the Covaris E220 sonicator (Covaris) and size selected using SPRISelect beads to 400 to 600 bp. The DNA was then enriched using the Arima-HiC v2 kit Enrichment beads. Using the NEBNext Ultra II DNA Library Prep Kit (New England Biolabs) for end repair, a-tailing, and adapter ligation. This uses a custom protocol which resembles the standard NEBNext Ultra II DNA Library Prep protocol but where library preparation occurs while DNA is bound to the Enrichment beads. For library amplification, 10 to 16 PCR cycles were required, determined by the sample biotinylation percentage. The Hi-C sequencing was performed using paired-end sequencing with a read length of 150 bp on an Illumina NovaSeq 6000 instrument.

***RNA***

Poly(A) RNA-Seq libraries were constructed using the NEB Ultra II RNA Library Prep kit, following the manufacturer’s instructions. RNA sequencing was performed on the Illumina NovaSeq X instrument.

### Genome assembly, curation and evaluation

***Assembly***

The HiFi reads were first assembled using Hifiasm ( Cheng *et al.*, 2021; Cheng *et al.*, 2022) in Hi-C phasing mode, resulting in a pair of haplotype-resolved assemblies. The Hi-C reads were mapped to the primary contigs using bwa-mem2 ( Vasimuddin *et al.*, 2019). The contigs were further scaffolded using the provided Hi-C data ( Rao *et al.*, 2014) in YaHS ( Zhou *et al.*, 2023) using the --break option for handling potential misassemblies. The scaffolded assemblies were evaluated using Gfastats ( Formenti *et al.*, 2022), BUSCO ( Manni *et al.*, 2021) and MERQURY.FK ( Rhie *et al.*, 2020).

The mitochondrial genome was assembled using MitoHiFi ( Uliano-Silva *et al.*, 2023), which runs MitoFinder ( Allio *et al.*, 2020) and uses these annotations to select the final mitochondrial contig and to ensure the general quality of the sequence.

***Assembly curation***

The assembly was decontaminated using the Assembly Screen for Cobionts and Contaminants (ASCC) pipeline (article in preparation). Flat files and maps used in curation were generated in TreeVal ( Pointon *et al.*, 2023). Manual curation was primarily conducted using PretextView ( Harry, 2022), with additional insights provided by JBrowse2 ( Diesh *et al.*, 2023) and HiGlass ( Kerpedjiev *et al.*, 2018). Scaffolds were visually inspected and corrected as described by Howe *et al*. (2021). Any identified contamination, missed joins, and mis-joins were corrected, and duplicate sequences were tagged and removed. The curation process is documented at https://gitlab.com/wtsi-grit/rapid-curation (article in preparation).

***Assembly quality assessment***

The Merqury.FK tool ( Rhie *et al.*, 2020), run in a Singularity container ( Kurtzer *et al.*, 2017), was used to evaluate *k*-mer completeness and assembly quality for the primary and alternate haplotypes using the *k*-mer databases ( *k* = 31) that were computed prior to genome assembly. The analysis outputs included assembly QV scores and completeness statistics.

A Hi-C contact map was produced for the final version of the assembly. The Hi-C reads were aligned using bwa-mem2 ( Vasimuddin *et al.*, 2019) and the alignment files were combined using SAMtools ( Danecek *et al.*, 2021). The Hi-C alignments were converted into a contact map using BEDTools ( Quinlan & Hall, 2010) and the Cooler tool suite ( Abdennur & Mirny, 2020). The contact map is visualised in HiGlass ( Kerpedjiev *et al.*, 2018).

The blobtoolkit pipeline is a Nextflow ( Di Tommaso *et al.*, 2017) port of the previous Snakemake Blobtoolkit pipeline ( Challis *et al.*, 2020). It aligns the PacBio reads in SAMtools and minimap2 ( Li, 2018) and generates coverage tracks for regions of fixed size. In parallel, it queries the GoaT database ( Challis *et al.*, 2023) to identify all matching BUSCO lineages to run BUSCO ( Manni *et al.*, 2021). For the three domain-level BUSCO lineages, the pipeline aligns the BUSCO genes to the UniProt Reference Proteomes database ( Bateman *et al.*, 2023) with DIAMOND blastp ( Buchfink *et al.*, 2021). The genome is also divided into chunks according to the density of the BUSCO genes from the closest taxonomic lineage, and each chunk is aligned to the UniProt Reference Proteomes database using DIAMOND blastx. Genome sequences without a hit are chunked using seqtk and aligned to the NT database with blastn ( Altschul *et al.*, 1990). The blobtools suite combines all these outputs into a blobdir for visualisation.

The blobtoolkit pipeline was developed using nf-core tooling ( Ewels *et al.*, 2020) and MultiQC ( Ewels *et al.*, 2016), relying on the Conda package manager, the Bioconda initiative ( Grüning *et al.*, 2018), the Biocontainers infrastructure ( da Veiga Leprevost *et al.*, 2017), as well as the Docker ( Merkel, 2014) and Singularity ( Kurtzer *et al.*, 2017) containerisation solutions.

Table 4 contains a list of relevant software tool versions and sources.

**Table 4.** Software tools: versions and sources.

Software tool	Version	Source
BEDTools	2.30.0	https://github.com/arq5x/bedtools2
BLAST	2.14.0	ftp://ftp.ncbi.nlm.nih.gov/blast/executables/blast+/
BlobToolKit	4.3.7	https://github.com/blobtoolkit/blobtoolkit
BUSCO	5.4.3 and 5.5.0	https://gitlab.com/ezlab/busco
bwa-mem2	2.2.1	https://github.com/bwa-mem2/bwa-mem2
Cooler	0.8.11	https://github.com/open2c/cooler
DIAMOND	2.1.8	https://github.com/bbuchfink/diamond
fasta_windows	0.2.4	https://github.com/tolkit/fasta_windows
FastK	427104ea91c78c3b8b8b49f1a7d6bbeaa869ba1c	https://github.com/thegenemyers/FASTK
Gfastats	1.3.6	https://github.com/vgl-hub/gfastats
GoaT CLI	0.2.5	https://github.com/genomehubs/goat-cli
Hifiasm	0.19.8-r603	https://github.com/chhylp123/hifiasm
HiGlass	44086069ee7d4d3f6f3f0012569789ec138f42b84 aa44357826c0b6753eb28de	https://github.com/higlass/higlass
Merqury.FK	d00d98157618f4e8d1a9190026b19b471055b22e	https://github.com/thegenemyers/MERQURY.FK
MitoHiFi	3	https://github.com/marcelauliano/MitoHiFi
MultiQC	1.14, 1.17, and 1.18	https://github.com/MultiQC/MultiQC
NCBI Datasets	15.12.0	https://github.com/ncbi/datasets
Nextflow	23.04.0-5857	https://github.com/nextflow-io/nextflow
PretextView	0.2	https://github.com/sanger-tol/PretextView
purge_dups	1.2.5	https://github.com/dfguan/purge_dups
samtools	1.16.1, 1.17, and 1.18	https://github.com/samtools/samtools
sanger-tol/ascc	-	https://github.com/sanger-tol/ascc
Seqtk	1.3	https://github.com/lh3/seqtk
Singularity	3.9.0	https://github.com/sylabs/singularity
TreeVal	1.0.0	https://github.com/sanger-tol/treeval
YaHS	1.2a.2	https://github.com/c-zhou/yahs

### Wellcome Sanger Institute – Legal and Governance

The materials that have contributed to this genome note have been supplied by a Darwin Tree of Life Partner. The submission of materials by a Darwin Tree of Life Partner is subject to the **‘Darwin Tree of Life Project Sampling Code of Practice’**, which can be found in full on the Darwin Tree of Life website here. By agreeing with and signing up to the Sampling Code of Practice, the Darwin Tree of Life Partner agrees they will meet the legal and ethical requirements and standards set out within this document in respect of all samples acquired for, and supplied to, the Darwin Tree of Life Project.

Further, the Wellcome Sanger Institute employs a process whereby due diligence is carried out proportionate to the nature of the materials themselves, and the circumstances under which they have been/are to be collected and provided for use. The purpose of this is to address and mitigate any potential legal and/or ethical implications of receipt and use of the materials as part of the research project, and to ensure that in doing so we align with best practice wherever possible. The overarching areas of consideration are:

•   Ethical review of provenance and sourcing of the material

•   Legality of collection, transfer and use (national and international)

Each transfer of samples is further undertaken according to a Research Collaboration Agreement or Material Transfer Agreement entered into by the Darwin Tree of Life Partner, Genome Research Limited (operating as the Wellcome Sanger Institute), and in some circumstances other Darwin Tree of Life collaborators.

## Data Availability

European Nucleotide Archive: Branchiostoma lanceolatum (amphioxus). Accession number PRJEB74948; https://identifiers.org/ena.embl/PRJEB74948. The genome sequence is released openly for reuse. The *Branchiostoma lanceolatum* genome sequencing initiative is part of the Darwin Tree of Life (DToL) project. All raw sequence data and the assembly have been deposited in INSDC databases. The genome will be annotated using available RNA-Seq data and presented through the Ensembl pipeline at the European Bioinformatics Institute. Raw data and assembly accession identifiers are reported in Table 1 and Table 2.

## Author information

Members of the Marine Biological Association Genome Acquisition Lab are listed here: https://doi.org/10.5281/zenodo.8382513.

Members of the Darwin Tree of Life Barcoding collective are listed here: https://doi.org/10.5281/zenodo.12158331.

Members of the Wellcome Sanger Institute Tree of Life Management, Samples and Laboratory team are listed here: https://doi.org/10.5281/zenodo.12162482.

Members of Wellcome Sanger Institute Scientific Operations: Sequencing Operations are listed here: https://doi.org/10.5281/zenodo.12165051.

Members of the Wellcome Sanger Institute Tree of Life Core Informatics team are listed here: https://doi.org/10.5281/zenodo.12160324.

Members of the Tree of Life Core Informatics collective are listed here: https://doi.org/10.5281/zenodo.12205391.

Members of the Darwin Tree of Life Consortium are listed here: https://doi.org/10.5281/zenodo.4783558.
