# Supplementary material for: The genome sequence of the amphioxus, Branchiostoma lanceolatum (Pallas, 1774)
Source: Wellcome Open Res. 2025 Feb 24;10:95. [Version 1] doi: 10.12688/wellcomeopenres.23671.1 (PMC11907188; doi:10.12688/wellcomeopenres.23671.1)
Supplement: Peer review history

# Reviewer report: Reviewer response for version 1

**DOI:** 10.21956/wellcomeopenres.26114.r119573

**Published:** 2025-04-04

**Authors:** Kun Wang

**Affiliations:** 1 School of Ecology and Environment, Northwestern Polytechnical University, Xi’an, China

The manuscript is exceptionally well-written and clearly organized, with a logical flow that effectively guides readers through the research. The high-quality genome assembly presented here represents a valuable contribution to the scientific community, particularly for researchers studying cephalochordate evolution and population genetics in northern European waters. The methodological approach is comprehensively described, with appropriate technical details that would enable reproducibility. The authors have done an excellent job contextualizing their findings within existing literature, particularly in highlighting the significance of comparing Atlantic and Mediterranean *B. lanceolatum* populations.

Are sufficient details of methods and materials provided to allow replication by others?

Yes

Is the rationale for creating the dataset(s) clearly described?

Yes

Are the datasets clearly presented in a useable and accessible format?

Yes

Are the protocols appropriate and is the work technically sound?

Yes

Reviewer Expertise:

Comparative genomics, vertebrate evolution

I confirm that I have read this submission and believe that I have an appropriate level of expertise to confirm that it is of an acceptable scientific standard.

# Reviewer report: Reviewer response for version 1

**DOI:** 10.21956/wellcomeopenres.26114.r119576

**Published:** 2025-03-18

**Authors:** Simona Candiani

**Affiliations:** 1 Dipartimento di Scienze della Terra, dell'Ambiente e della Vita, Università di Genova, Genoa, Italy

The paper by Adkins and colleagues presents a data note on the genome sequence of a female *Branchiostoma lanceolatum* amphioxus collected in Devon, UK. This represents the first genome sequencing effort for an Atlantic population of this species.

 Additionally, the mitochondrial sequence and transcriptomic data from muscle tissue have also been obtained.

 These new data will be valuable for future annotation efforts, potentially enabling the community to generate new amphioxus genomes with improved annotations compared to previous versions.

 In this regard, it would be interesting if the authors included a few lines discussing the quality of this sequencing compared to previous efforts for *Branchiostoma lanceolatum (Marletz et al., 2018; Simakov et al., 2020)*.

 Overall, the report notes are well-written, and the technical procedures are clearly described.

Are sufficient details of methods and materials provided to allow replication by others?

Yes

Is the rationale for creating the dataset(s) clearly described?

Yes

Are the datasets clearly presented in a useable and accessible format?

Yes

Are the protocols appropriate and is the work technically sound?

Yes

Reviewer Expertise:

Developmental neuroscience and comparative genomics

I confirm that I have read this submission and believe that I have an appropriate level of expertise to confirm that it is of an acceptable scientific standard.

## References

[1] : Amphioxus functional genomics and the origins of vertebrate gene regulation. *Nature* .2018;564(7734) : 10.1038/s41586-018-0734-6 64-70 10.1038/s41586-018-0734-6 30464347 PMC6292497

[2] : Deeply conserved synteny resolves early events in vertebrate evolution. *Nat Ecol Evol* .2020;4(6) : 10.1038/s41559-020-1156-z 820-830 10.1038/s41559-020-1156-z 32313176 PMC7269912

# Reviewer report: Reviewer response for version 1

**DOI:** 10.21956/wellcomeopenres.26114.r119572

**Published:** 2025-03-17

**Authors:** Jr-Kai Yu

**Affiliations:** 1 Academia Sinica, Taipei, Taiwan

In this paper, the authors report a new complete genome sequence and RNA-seq dataset from one single female adult of the amphioxus *Branchiostoma lanceolatum* collected from coastal sediments off Devon, UK. This new genome assembly will complement well to the existing genome dataset of *B. lanceolatum* collected from the Mediterranean population, and will be very useful for future comparative genomic studies with other cephalochordate species or with other groups of animals. Sufficient details of the methods and materials are provided in this paper, and the work flow and protocols appear to follow the standard operating procedures of a genome sequencing project. All raw sequence data and the assembly have been deposited in appropriate public domain.

**I only have the following minor comments:**

1. Regarding the RNA-seq dataset, in this paper the RNA was extracted only from the muscle tissue of the same adult sample. I am not sure whether this is because the standard procedure of the Darwin Tree of Life project requires the RNA sample to be collected from the same type of tissue for DNA sequencing. Generally, the muscle tissue contains a less diverse transcriptome profile and may not be able to provide an overall view of the expressed genes in an organism. If possible, collecting more tissue types, or some mixed-tissue type samples (such as cutting the entire animal into four pieces for RNA extraction individually) for RNA-seq may provide a more comprehensive dataset for analyzing the transcrioptome of this sample.
2. Page 10, 2 ^nd^ paragraph of the right column, 8 ^th^ line of this paragraph, the word “pipelinealigns” should be “pipeline aligns”.
3. Page 12, the reference list for “Brasó-Vives M, Marlétaz F, Echchiki A: Parallel evolution of amphioxus and…”; “ *et al.*” should be added after the name “Echchiki A”, because there are more than three authors of this paper.

In addition, I notice that in previous Reviewer Report by Luohao Xu (posted on 27 February 2025, https://doi.org/10.21956/wellcomeopenres.26114.r119574), it was stated that “A *B. lanceolatum* genome has also been published by Simakov et al.(2020).” Actually, the Simakov et al. (2020) paper is on the chromosome-scale genome assembly of the Florida amphioxus *B. floridae*, not *B. lanceolatum*.

Are sufficient details of methods and materials provided to allow replication by others?

Yes

Is the rationale for creating the dataset(s) clearly described?

Yes

Are the datasets clearly presented in a useable and accessible format?

Yes

Are the protocols appropriate and is the work technically sound?

Yes

Reviewer Expertise:

Evolutionary Developmental Biology, Comparative Genomics, Chordate Evolution

I confirm that I have read this submission and believe that I have an appropriate level of expertise to confirm that it is of an acceptable scientific standard.

# Reviewer report: Reviewer response for version 1

**DOI:** 10.21956/wellcomeopenres.26114.r119571

**Published:** 2025-03-17

**Authors:** Salvatore D'Aniello

**Affiliations:** 1 Stazione Zoologica Anton Dohrn, Napoli, Italy

The report by Adkins and collaborators reports the complete genome sequence and assembly of *Branchiostoma lanceolatum* (Pallas, 1774) collected from Devon, UK. The Tree of Life consortium methodologies adhere to best genome assembly practices, and in particular, the use of PacBio HiFi long-read sequencing and Hi-C chromosome conformation capture have enabled the authors to achieve a high-quality, chromosome-level assembly. This resource will be highly valuable for comparative genomic analysis between *Branchiostoma lanceolatum* Atlantic and Mediterranean populations, a comparison that was previously not possible due to the availability of high-quality genome resources only from specimens collected in the Mediterranean Sea (southern France). This additional amphioxus genome will also be useful in future studies aimed at determining whether slight morphological differences among European amphioxus populations, as well as variations in breeding periods and developmental rates, could have a genetic or environmental basis.

 The rationale is clear, the datasets are accessible, and I have no major comments or concerns.

Are sufficient details of methods and materials provided to allow replication by others?

Yes

Is the rationale for creating the dataset(s) clearly described?

Yes

Are the datasets clearly presented in a useable and accessible format?

Yes

Are the protocols appropriate and is the work technically sound?

Yes

Reviewer Expertise:

Evolution and Developmental Biology

I confirm that I have read this submission and believe that I have an appropriate level of expertise to confirm that it is of an acceptable scientific standard.

# Reviewer report: Reviewer response for version 1

**DOI:** 10.21956/wellcomeopenres.26114.r119577

**Published:** 2025-03-13

**Authors:** Guang Li

**Affiliations:** 1 Xiamen University, Xiamen, China

Complementary to previous studies that presented the genome sequences of a male and a gender-unknown *B. lanceolatum* from the Mediterranean sea, this study report the genome sequence of a female *B. lanceolatum* collected from coastal sediments off Devon, UK. It will be a valuable resource for research aiming to investigate population genetics and local adaptations of this lancelet species as the authors stated.  I have several minor comments on it as following:

 1) It would be useful to provide the quality information of the two haplotypes after initial assembly (before manual curation).  

 2) It would be better to offer an equivalent information for the haplotype 2 assembly as that described in the 3rd paragraph for the haplotype 1 in the report section.

 3) Some comparisons between the assembly reported here and the one presented by  Brasó-Vives et al. (2022) should be conducted. 

 4)  Please make sure panel b in Figure 1 was taken from the same individual as panel a.

Are sufficient details of methods and materials provided to allow replication by others?

Yes

Is the rationale for creating the dataset(s) clearly described?

Yes

Are the datasets clearly presented in a useable and accessible format?

Yes

Are the protocols appropriate and is the work technically sound?

Yes

Reviewer Expertise:

evolutionary and developmental biology

I confirm that I have read this submission and believe that I have an appropriate level of expertise to confirm that it is of an acceptable scientific standard.

# Reviewer report: Reviewer response for version 1

**DOI:** 10.21956/wellcomeopenres.26114.r119574

**Published:** 2025-02-27

**Authors:** Luohao Xu

**Affiliations:** 1 MOE Key Laboratory of Freshwater Fish Reproduction and Development, School of Life Sciences, Southwest University, Chongqing, China

Please make sure that the taxon information is corrrect: *Branchiostoma lanceolatum* (Amphioxus; Chordata; Leptocardii; Amphioxiformes; Branchiostomatidae)

 The background part failed to cover more recent study using single-cell technology on amphioxus, such as Dai Y *et al.*(2024), Wu B *et al.*(2024) and Ma P *et al.* (2022)

 A B. lanceolatum genome has also been published by Simakov *et al.(*2020). Please also include comparisons with other *Branchiostoma *genomes: Huang Z *et al.(2023)*

Are sufficient details of methods and materials provided to allow replication by others?

Yes

Is the rationale for creating the dataset(s) clearly described?

Yes

Are the datasets clearly presented in a useable and accessible format?

Yes

Are the protocols appropriate and is the work technically sound?

Yes

Reviewer Expertise:

genomics

I confirm that I have read this submission and believe that I have an appropriate level of expertise to confirm that it is of an acceptable scientific standard.

## References

[1] : Evolutionary origin of the chordate nervous system revealed by amphioxus developmental trajectories. *Nat Ecol Evol* .2024;8(9) : 10.1038/s41559-024-02469-7 1693-1710 10.1038/s41559-024-02469-7 39025981

[2] : Single-cell analysis of the amphioxus hepatic caecum and vertebrate liver reveals genetic mechanisms of vertebrate liver evolution. *Nat Ecol Evol* .2024;8(10) : 10.1038/s41559-024-02510-9 1972-1990 10.1038/s41559-024-02510-9 39152328

[3] : Joint profiling of gene expression and chromatin accessibility during amphioxus development at single-cell resolution. *Cell Rep* .2022;39(12) : 10.1016/j.celrep.2022.110979 110979 10.1016/j.celrep.2022.110979 35732129

[4] : Deeply conserved synteny resolves early events in vertebrate evolution. *Nat Ecol Evol* .2020;4(6) : 10.1038/s41559-020-1156-z 820-830 10.1038/s41559-020-1156-z 32313176 PMC7269912

[5] : Three amphioxus reference genomes reveal gene and chromosome evolution of chordates. *Proc Natl Acad Sci U S A* .2023;120(10) : 10.1073/pnas.2201504120 e2201504120 10.1073/pnas.2201504120 36867684 PMC10013865
